# New York Academy of Medicine—Section on Orthopedic Surgery

**Published:** 1891-02

**Authors:** V. P. Gibney

**Affiliations:** Chairman


### NEW YORK ACADEMY OF MEDICINE.—SECTION ON ORTHOPEDIC SURGERY.

STATED Meeting November 21, 1890.

V. P. GIBNEY, M. D., Chairman.

### DR. BEELY'S APPARATUS FOR MEASURING THE THORAX.

Dr. N. M. SHAFFER exhibited one of these instruments which had been presented to him while he was in Berlin, by the inventor, Dr. Beely. A somewhat similar apparatus has been called by Dr. Nebel, a kyrtograph. The apparatus consists of a series of narrow parallel steel bars, placed closely together, and sliding in a rectangular metal frame. Each bar terminates in a blunt point, near the end of which, on the under surface, is a metallic point or stylus. This arrangement of bars looks not unlike a huge comb. On bringing the pointed ends against the chest, or any similar object having an irregular outline, the bars adjust themselves in the frame, so that the pointed ends form an outline identical with that of the object against which they were placed. A simple cam movement then clamps them in this position. The instrument is next laid upon a piece of paper, resting on a sheet of felt, and the points on the under surfaces of the bars are made to puncture the paper, by passing a small roller over all the bars successively. In this way, the desired outline is recorded on paper as a series of small punctures, about one-fifth of an inch apart. When taking a tracing from a patient while standing, an erect position is secured by means of a plumb-line fastened to a belt, which is buckled around the body. In order to secure still greater accuracy, the instrument is provided with a spirit level. The exact level at which this transverse tracing of the chest is taken, may be marked with nitrate of silver, so that tracings taken at different times may be readily compared. With the patient in the prone position, the instrument may be used ; but a longitudinal tracing, as of the spinal column, with the patient erect, is not within the contemplated scope of the instrument.

Dr. Shaffer said that he had made several tracings with this instrument, and he had been very favorably impressed with its action and accuracy.

Dr. John Ridlon said that he had seen Dr. Beely using it with the patient in the prone position, and that he had understood that its action was only satisfactory when employed in this way. At the Berlin congress, Dr. Nebel had exhibited a much cheaper, but less accurately made instrument, constructed on the same principle. A series of round rods were placed parallel to each other, and made to slide through a large round bar. The small bars terminated in buttons, which were applied to the chest. The outline was then transferred to paper by laying the machine on the paper, and passing a pencil along the buttons.

Dr. A. B. Judson admired the mechanical features of the instrument, but thought that such tracings of cases of lateral curvature were of but little value, in view of the fact that the changes in contour occurred from day to day, as a result of differences in the general tone of the patient.

### A CASE OF SUPPOSED EVACUATION OF A PSOAS, OR LUMBAR ABSCESS THROUGH THE VAGINA.

Dr. Samuel Ketch presented such a case. A child, five and three-quarter years of age, of healthy parentage, and having a good family history, was admitted to the hospital on October 22, 1890, with the history of a fall six or eight weeks prior to her admission. Two weeks before she was presented for examination, she was first noticed to walk peculiarly, and she complained of "pain in her feet." She was thought to be suffering from hip-joint disease by her attending physician. At the time of the examination, her general condition was fair, and there was no pain, and the motions at the hip-joint were normal with the exception of a slight limitation of extension on the right side, when the patient was in the prone position. Close examination revealed a small kyphos in the lower lumbar region; the right hip was prominent and relatively larger, and there was a feeling of fluctuation on that side. There was a discharge of pus from the vagina. The case was of interest, both on account of the unusual mode of evacuation of the pus, and because of the symptoms which had led to the diagnosis of hip-joint disease.

Dr. Shaffer had seen the case just reported. He had met with cases where the abscesses of Pott's disease opened into the rectum, vagina, and bladder; and in one case of disease in the dorso-lumbar region, which had extended over a period of about twenty years, pus was discharged from the bladder at intervals of a few months, accompanied by the usual symptoms of acute abscess formation. The cases in which the abscess had opened in these unusual situations, had all done well, and he attributed this to the valvular opening through which the pus was discharged. In one of his cases, an abscess was found pointing into the rectum, and was purposely evacuated at this point by means of a trocar. The result had been extremely satisfactory. A similarly favorable prognosis could not be given where abscesses ruptured into the lungs. He had seen several of these cases, and in one now under observation, which he considered unique, a boy with mid-dorsal disease, suffered at intervals from fever, accompanied by the expectoration of pieces of the cancelous portion of bone.

Dr. R. H. Sayre said that anatomical considerations would lead one to expect that owing to better drainage, abscesses opening into the alimentary canal would pursue a more favorable course than those which ruptured into the lung. He recalled a case where an abscess, situated on each side of the vertebræ, suddenly enlarged and caused fatal asphyxia. The autopsy showed that "a saddle-bag" abscess was situated at the bifurcation of the trachea.

Dr. J. K. Young, of Philadelphia, said that he had recently seen a case of lumbar Pott's disease, in which the abscess had been evacuated through the vagina, and also through the abdominal walls. It had pursued a favorable course.

Dr. Thomas H. Manley had once treated a case of abscess in the mid-dorsal region, where evacuation had occurred through the umbilicus. It made a good recovery.

Dr. Agramonte suggested that the exact condition present in Dr. Ketch's case could not be affirmed until the vaginal discharge had been examined for gonococci.

The Chairman thought that such an examination was highly important, and suggested that search should also be made for the opening through which the pus was discharged.

Dr. Ketch, in closing the discussion, said that he was aware that the report of the case was somewhat premature, and had only been presented at the request of the Chairman for clinical material. He had not been able as yet to make the examinations suggested ; but the rational symptoms and the clinical history of the case seemed to warrant the position which he had taken. In an experience of thirteen years at the Orthopedic Dispensary, he had never before met with such a case, and had only seen one case in private practice, and in this one, Dr. L. A. Sayre had verified the condition. In this case there was a favorable termination.

